# Tremellomycetes Yeasts in Kernel Ecological Niche: Early Indicators of Enhanced Competitiveness of Endophytic and Mycoparasitic Symbionts against Wheat Pathobiota

**DOI:** 10.3390/plants10050905

**Published:** 2021-04-30

**Authors:** Vladimir Vujanovic

**Affiliations:** Department of Food and Bioproduct Sciences, University of Saskatchewan, 51 Campus Drive, Saskatoon, SK S7N 5A8, Canada; vladimir.vujanovic@usask.ca

**Keywords:** Tremellomycetes, Triticale, wheat, biocontrol, ITS rDNA, endophyte, mycoparasite

## Abstract

Tremellomycetes rDNA sequences previously detected in wheat kernels by MiSeq were not reliably assigned to a genus or clade. From comparisons of ribosomal internal transcribed spacer region (ITS) and subsequent phylogenetic analyses, the following three basidiomycetous yeasts were resolved and identified: *Vishniacozyma* *victoriae*, *V. tephrensis,* and an undescribed *Vishniacozyma* rDNA variant. The *Vishniacozyma* variant’s clade is evolutionarily close to, but phylogenetically distinct from, the *V. carnescens* clade. These three yeasts were discovered in wheat kernel samples from the Canadian prairies. Variations in relative *Vishniacozyma* species abundances coincided with altered wheat kernel weight, as well as host resistance to chemibiotrophic *Tilletia* (Common bunt—CB) and necrotrophic *Fusarium* (Fusarium head blight—FHB) pathogens. Wheat kernel weight was influenced by the coexistence of *Vishniacozyma* with endophytic plant growth-promoting and mycoparasitic biocontrol fungi that were acquired by plants. Kernels were coated with beneficial *Penicillium* endophyte and *Sphaerodes* mycoparasite, each of which had different influences on the wild yeast population. Its integral role in the kernel microbiome renders *Vishniacozyma* a measurable indicator of the microbiome–plant interaction. The ability of NGS technology to detect specific endophytic DNA variants and early changes in dynamics among symbionts within the kernel ecological niche enables the prediction of crop disease emergence, suggesting that advanced microbiological testing may be a potentially useful tool for both phytoprotection and more efficient wheat breeding programs.

## 1. Introduction

The seed, grain, or kernel is the reproductive unit of plants [1]. In wild as well as domesticated *Triticale* plants, kernels simultaneously establish multiple symbiotic associations with endophytic and mycoparasitic fungi, which can, respectively, improve plant traits and increase resistance to pathogenic fungi. Transgenerational transmission of the kernel mycobiome composition has been reported to be a vital prerequisite for stress resilience and adaptive phenotypes in *Triticum* [2]. However, the numerous fungal interrelationships that exist within grains, particularly those between beneficial fungi and pathobiota affecting competitive plant traits, remain poorly understood. Given that the health of plants depends on the health of the kernels from which they develop, it is important to understand the role of mycovitality, which is the relationship between the grain and endophytic microbes [3,4,5,6] that acts as a key regulatory mechanism affecting host physiology and phenotypic characteristics. Understanding this relationship is a critical first step toward enhancing developmental events and early resilience to environmental stressors, including pathobiota, in the plant host and is a cornerstone of pre-germination care [7].

In a long-term project designed to examine the ecology of the fungal microbiome within asymptomatic wheat kernels, Tremellomycetes (Basidiomycota) yeast endosymbionts were discovered in high proportions in samples grown in the Canadian prairies [2]. Among fungal endophytic communities, US scientists discovered yeasts in the phyllospheres of maize (*Zea mays*) and northern wild rice (*Zizania palustris*) growing in the fields of Minnesota and Illinois [8]. Although nearly 500 basidiomycetous yeasts have been widely documented, only a few psychrophilic *Vishniacozyma* and *Rhodotorula* yeasts have been discovered in northern regions, glacial mountains, and polar habitats [9,10]. Among cold-adapted endophytic fungi, *Vishniacozyma*, *Cryptococcus,* *Rhodotorula*, and *Aureobasidium* yeasts formed a complex assemblage with biocontrol antagonists, such as *Cladosporium uredinicola* and *Coprinellus micaceus*, endophytic generalists (e.g., *Alternaria* and *Penicillium*), and plant pathogens (e.g., *Cercospora* and *Myrothecium*) discovered in whet spikes and medicinal plants [11,12]. Based on GC–MS spectra analysis, *Vishniacozyma victoriae* within Tremellales produces a diverse range of bioactive metabolites [12] and was a subject of the diversity bioprospecting of antimicrobial metabolites [13] and enzymes [14]. It has been suggested that the phyllosphere-associated pigmented yeasts of genera *Vishniacozyma*, *Rhodotorula*, and *Sporoblomyces* are helping plant growth and ecological adaptation to cold environments [15] by synthesizing substantial amounts of auxin and zeatin [16,17,18].

Yeasts in the order Tremellales are integral components of fungal communities in soil rhizosphere/root, phyllosphere/stem and leaf [1], and semosphere/kernel [2]. Tremellales, known as ‘jelly fungi’, are predominantly mycoparasitic or fungicolous and grow on a wide range of basidiomycetous and ascomycetous fungi [19,20,21,22]. Certain Tremellales taxa, however, are highly host-specific and are frequently confined to a single fungal genus or species. Thus, many tremellaceous yeasts that had previously been classified as *Cryptococcus* are now incorporated into newly described genera [23,24]. One such newly established genus is *Vishniacozyma*, which currently consists of 23 taxa, according to Kirk [25], with several erected species: *Vishniacozyma alagoana, V. carnescens*, *V. dimennae*, *V. europaea*, *V. ellesmerensis, V. foliicola, V. globispora, V. heimaeyensis, V. kurzmanii, V. melezitolytica*, *V. nebularis, V. penaeus, V. phoenicis, V. pseudopenaeus, V. psychrotolerans, V. taibaiensis, V. tephrensis*, and *V. victoriae* [10,23,26]. Though the range of plant–yeast interactions includes parasitism against hemibiotrophic (*Tilletia, Taphrina*, and *Ustilago*) pathogens [8], the possible roles of *Vishniacozyma* as an endophyte or a mycoparasite remain unexplored [11]. While the molecular mechanism of fungus–host recognition is still unknown, the mycoparasitic nature of yeasts is related to their anamorphic or dikaryotic stage-associated infective structures, such as tremelloid-specialized haustoria [22]. In addition to potential biological control and commercial applications [27], the biotechnological potential of *Vishniacozyma* is linked to the biosynthesis of yeast oils [28], as well as the carbon-active and proteolytic enzymes, which are regulated by numerous genes [14]. Further, yeasts have been tested for plant protection as antagonists of common fungal plant pathogens in the management of postharvest diseases and for the biological decontamination of agricultural products from mycotoxins [8]. However, the relationship between Tremellales and its wheat host is still unknown. Hence, the beneficial endophytic and mycoparasitic yeast features merit further investigation focusing on both individual fungus–fungus as well as fungus–microbiome interaction levels in specific yeast–host systems and environments.

While several of the Tremellales yeasts are recognized mycoparasites that may control *Tilletia*, Common bunt (CB), and Smut diseases, *Sphaerodes mycoparasitica* is a biotrophic mycoparasite specific to *Fusarium graminearum,* which causes Fusarium head blight (FHB) in small cereal grains [29]. Both types of mycoparasitism were previously discovered in wheat kernels [2,30]. Since *Tremellales* mycoparasites tend toward plant–host specificity, the present study has focused on the identification of uncharacterized yeast populations in wheat kernels from previous field and current greenhouse samplings. Next-generation MiSeq DNA sequencing (NGS) analyses were used to identify yeasts, in addition to assessing their interactions with other endophytic and parasitic fungi in wheat. 

In this study, we specifically tested whether wheat G_0_ (mother plant)-kernel coating with functionally different, plant growth-promoting (PGP) *Penicillium* sp. and *Sphaerodes mycoparasitica* endophytes could alter plant traits and/or modulate competition between naturally occurring *Tremellales* yeasts and two coexistent fungi, *Tilletia* (Basidiomycota) and *Fusarium* (Ascomycota), in G_1_ (first generation) kernels. The question was raised of whether the abundance of Tremellales yeasts within the G_1_ ecological niche (based on NGS analysis) is affected differently by the application of *Penicillium* sp.-PGP versus *Sphaerodes* mycoparasite–BCA inoculants. Although *Tremellales* and *Sphaerodes* mycoparasites may belong to the same trophic guild within a plant endosphere, such as the wheat kernel ecological niche, their specificity towards chemobiotrophic (*Tilletia*) versus necrotrophic (*Fusarium*) pathogens may have different effects on wheat crop resistance and crop production traits.

## 2. Materials and Methods

### 2.1. Plant and Fungal Materials

This study measured the respective effects of *Penicillium* sp. SMCD 2318 endophyte [2] and *Sphaerodes mycoparasitica* SMCD 2220 mycoparasite [30,31] strains on the diversity and abundance of *Tremellales* yeasts, the harvested kernel weight/yield, and the level of suppression of *Fusarium* (Fusarium head blight—FHB) and *Tilletia* (Common bunt—CB) pathogens. Sampling, treatment, and DNA extraction of yeasts followed previously described protocols [2]. In short, the Illumina-MiSeq next-generation sequencing (NGS) of ribosomal internal transcribed spacer region (ITS) was used to identify *Tremellales* taxa in surface-sterilized kernels of AC Avonlea wheat (*Triticum turgidum* L. var. durum) from plants grown in a CONVIRON ^®^ PGR15 growth chamber (Controlled Environments Ltd., Winnipeg, MB, Canada). The control and pre-inoculated kernels (G_0_) were coated with SMCD 2318 endophyte and SMCD 2220 mycoparasite, which were used to establish interspecific interactions in this experiment. The (G_0_) kernels were planted in 4 L plastic pots (three plants per pot) with autoclaved Sunshine Mix 4 (SunGro Horticulture Canada Ltd., Vancouver, BC, Canada). Each study group consisted of 5 replicates (15 plants per treatment). All potted plants were grown in separate, disinfected compartments inside the growth chambers, and were watered and fertilized as recommended. After approximately three months, the plant heads were assessed at the plant maturity stages GS 83 and GS 87 (based on Zadok’s growth scale) [32] for FHB and Common bunt incidence. The method for FHB and CB assessments was based on the sampling/scoring scales of 150 heads, as previously reported by Paul et al. [33] and Riccioni et al. [34]. Both the FHB and the CB indexes were calculated [incidence × severity)/100] for the treatment groups involving endophytic microbial bioinoculants [35]. 

### 2.2. DNA Isolation

The G_1_ kernels from harvested plants were surface-sterilized in 75% ethanol for 10 s, rinsed with sterile distilled water for 10 s, submerged for 3 min in 5% sodium hypochlorite (Javex ^®^ 12 Bleach), and then rinsed five times with sterile distilled water prior to DNA extraction [2]. The Illumina MiSeq library preparation and analyses were based on ITS 5.8S rRNA, as previously described in Vujanovic et al. [2].

The MiSeq Illumina DNA sequencing generated three Tremellales yeast operational taxonomic units (OTUs) or ITS sequence variants, while detected yeasts were further confirmed via re-isolation on YM agar medium [8] from surface-sterilized kernel samples [2] to specifically isolate the endophytic fungi. The relative abundance of the fungal OTUs classified as Tremellales yeasts was calculated as the percentage of sequence reads belonging to a specific OTU or rDNA variant in relation to the total number of reads in a sample. A radar chart of the relative abundance of the particular *Vishniacozyma* species in each treatment was created using Microsoft Excel version 2016. Based on the above data, the Yeast Early Index (YE_index_) was calculated (relative abundance × no. samples)/100. The term “Early” denotes that the yeast acts as an indicator of early plant protection based on the study of the kernel microbiome, as described within the context of the previously described “plant prenatal care” concept [7].

### 2.3. Phylogenetic Analysis

**Figure 1:**
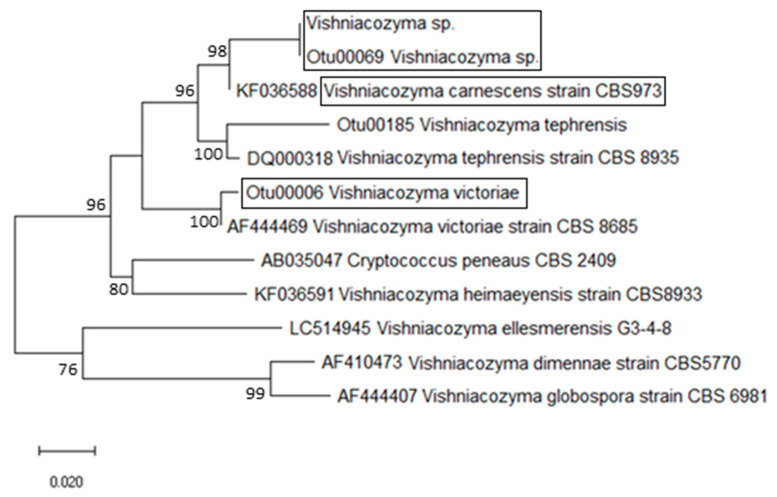
Phylogenetic tree of *Vishniacozyma/Cryptococcus* taxa constructed by using tremellomycetous ribosomal internal transcribed spacer region (ITS) sequences from wheat kernels (encircled in black boxes); sequences used here show >98% similarity with sequences deposited in the NCBI database (www.ncbi.nih.gov, accessed on 30 April 2021). The evolutionary history was inferred using the Mega X software; the optimal tree with the sum of branch length = 0.48037206 is shown. Bar, number of expected substitutions accumulated per site. Bootstrap values of ≥70% are shown (1000 replicates) on the nodes.

**Figure 2:**
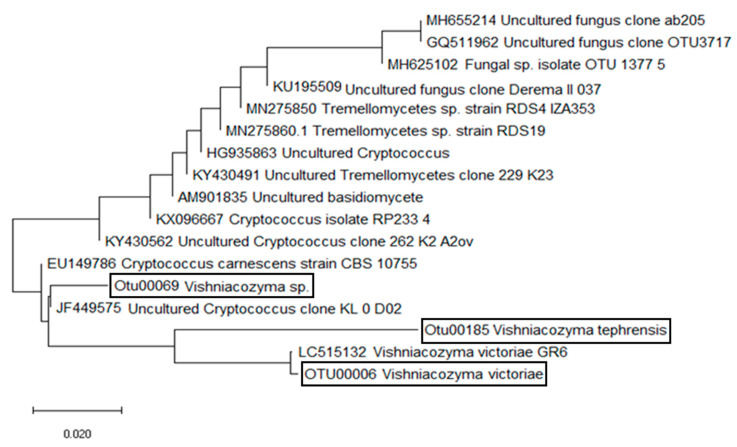
Unrooted tree showing the evolutionary relationship and phylogenetic placement of *Vishniacozyma/Cryptococcus* taxa discovered on wheat (encircled in black boxes). This tree is in agreement within the phylogenomic framework reported for Tremellomycetes by Liu et al. (2015) based on ITS sequences. The evolutionary history was inferred using the Mega X software, while the optimal tree with the sum of branch length = 0.22492958 is shown. Bar, number of expected substitutions accumulated per site.

The nucleotide sequences of three dominant yeasts were frequently detected in both untreated and treated wheat kernels. These ITS 5.8 rRNA sequences were checked against the NCBI (www.ncbi.nih.gov, accessed on 30 April 2021) databases. First, the NCBI sequences with >98% similarity were retrieved to perform phylogenetic and evolutionary analyses (Figure 1). The second set of *Vishniacozyma*/*Cryptococcus* sequences published by Liu et al. [23] was used to provide ITS reference sequences, allowing both phylogenetic validation and accurate classification of the three discovered Tremellomycetes yeasts (Figure 2). The evolutionary history was inferred using the minimum evolution method [36], while evolutionary distances were computed using the maximum composite likelihood method [37]. The ME tree was searched using the Close-Neighbor-Interchange (CNI) algorithm [38], while the neighbor-joining algorithm [39] was used to generate the phylogenetic tree. After all ambiguous positions were removed, evolutionary analyses were conducted in MEGA × [40]. Nucleotide sequences were deposited in NCBI/EMBL GenBank under the accession numbers that are provided in the text. 

### 2.4. Statistical Analysis

In the growth chamber, the harvested kernel weight/yield was evaluated as one of the plant traits more indicative of crop vigour and resilience to disease. The kernel data (g/pot) were statistically analyzed for all treatments, using one-way analysis of variance (ANOVA), to test the hypotheses that plant fitness may be influenced by *Penicillium*-endophyte (Endo = PGP) and *Sphaerodes*-mycoparasite (Endo-Myc). The ANOVA was also conducted on percentage values for DI, DS, and FHB and CB indexes for each individual treatment (Endo = PGP/Endo-BCA) and evaluated using the post hoc Tukey’s test with a level of significance of *p* ≤ 0.05 (SPSS Statistics 26.0, 2016).

## 3. Results

A total of 16,895 NGS-based ITS sequence reads representing core fungal endophytes in wheat kernels across all three treatments (control, *Penicillium*-endophyte (PGP), and *Sphaerodes*-mycoparasite (BCA)) were generated. Individual analyses of the fungal taxa showed an average of 43.1% reads from the class Dothideomycetes (e.g., Pleosporales: *Alternaria*) and 24.6% from Sordariomycetes (e.g., Hypocreales: *Fusarium*). Among Sordaryomycetes reads, 0.5% represented beneficial *Clonostachis/Trichoderma*/*Sphaerodes* mycoparasites. Other beneficial fungi included Tremellales, at 1.7% (Tremellomycetes), and Eurotiales, at 0.3% (Eurotomycetes), including genera from the family Trichocomaceae, such as *Penicillium*.

Based on molecular phylogenetic analysis of the ITS sequences, the two yeast taxa, presented in Figure 1, were classified as *Vishniacozyma* *victoriae* (M.J. Montes, Belloch, Galiana, M.D. García, C. Andrés, S. Ferrer, Torr.-Rodr. & J. Guinea) Xin Zhan Liu, F.Y. Bai, M. Groenew. & Boekhout (GenBank acc. number: MT452522) and *V. tephrensis* X.Z. Liu, F.Y. Bai, M. Groenew. & Boekhout (98.9% similarity to GenBank acc. no. KX096667 and 96.5% similarity to GenBank acc. no. DQ000318 for the CBS:8935 type strain). These Tremellales species, together with *V. carnescens* (Verona & Luchetti) X.Z. Liu, F.Y. Bai, M. Groenew. & Boekhout (species type), have been previously reported in the phylloplanes of maize and northern rice [8]. A third Tremellales yeast taxon was also discovered in this study; it is different from all other known *Vishniacozyma*/*Cryptococcus* yeasts, while showing 100% similarity to a yeast (GenBank acc. number: MT452521) previously detected on wheat [2]. The phylogenetic placement of *Vishniacozyma*/*Cryptococcus* taxa, shown in Figure 1, was validated when this tree was combined with the more integrated phylogenetic classification of Tremellales (Figure 2), which was based on the ITS reference sequences [23]. The presented ITS phylogeny supports the delineation of Tremellales yeast taxa within the phylogenomic framework for Tremellomycetes reported by Liu et al. [23,26].

Indeed, the two phylogenetic trees, shown in Figure 1 and Figure 2, showed similar topologies, depicting the *Vishniacozyma* MT452521 taxon as a distinct DNA variant from the sister clade of *V. carnescens* (type species; GenBank acc. number: DQ000308). *Vishniacozyma* species within Tremellales [41] mycoparasites form gelatinous basidiocarps, ellipsoidal basidiospores, and H-shaped conidia [23]. Found in kernel samples, the MT452521 taxon is infrequently associated with *Vishniacozyma* *victoriae* (GenBank acc. number: MT452522)*,* while frequently accompanied by *V. tephrensis* (99.6% similarity with GenBank acc. number: KU745369) and *Rhodosporidiobolus colostri* yeast (syn. *Rhodosporula colostri*; 99.6% similarity with GenBank acc. number: KU745369). The toponym *seminis* is proposed for this distinct *Vishniacozyma* MT452521 rDNA variant or taxon; the epithet “seminis” is derived from the Latin word “sēmen,” which denotes a kernel on a plant or a fertilized grain. 

**Figure 3:**
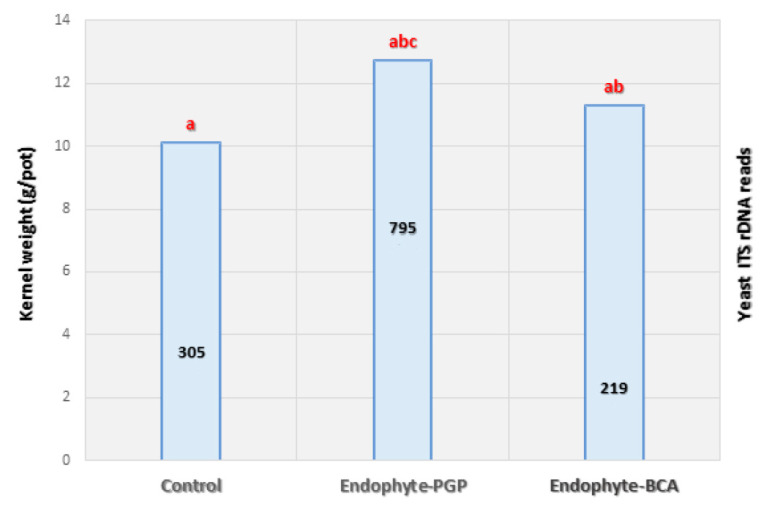
Wheat kernel weight/yield (g/plot) for endophytic *Penicillium* SMCD2318-plant growth promoter (PGP) versus *Sphaerodes* SMCD2220-biological control agent (BCA) treatments (left y-axis). Relative *Vishniacozyma* spp. abundance through treatments based on the ITS nucleic sequences reads by Illumina MiSeq technology (right y-axis). One-way analysis of variance (ANOVA) with Tukey’s test in post hoc was performed at *p*-value less than 0.05. Each bar represents five (three plants per pot) replicates under CONVIRON^®^ PGR15 growth chamber conditions. Values with different letters (**a**, **ab**, **abc**) on the histogram are significantly different (Tukay’s test, *p* < 0.05).

The results of the present study reveal that the abundance of *Vishniacozyma* spp. yeasts differed in the kernels of wheat plants inoculated with *Penicillium* and *Sphaerodes* (Figure 3). A considerable increase in yeast abundance was induced by *Penicillium*-endophyte (PGP), while *Sphaerodes*-mycoparasite (BCA) reduced the abundance of *Vishniacozyma* spp., possibly through the existence of interspecific competition (Figure 3). Variations in the presence of *Vishniacozyma* spp. correlated with increased kernel weight (Figure 3), ranging from +3.6 to + 7.5 g/pot when treated with *Sphaerodes* and *Penicillium*, respectively, compared to the control.

*Sphaerodes* inoculation resulted in a significant reduction in FHB incidence (~85%) and the closely related FHB Index (2.9 → 0.1; Table 1), which takes yeast abundance (~30%) into account. The YE_index_ was reduced from 0.35 to 0.20 in kernel samples (Figure 3). In contrast to the suppressive effect of BCA on yeasts, *Penicillium* treatment coincided with increased (~40%) *Vishniacozyma* spp. abundance compared to the control, and an increase in the YE_index_ from 0.35 to 0.55, which also coincided with suppressed (~80%) *Tilletia* incidence and a related decrease in the CB Index (3.1 → 0.4; Table 1). The latter indicates that the *Penicillium*–*Vishniacozyma* consortium may specifically control CB, while *Sphaerodes* specifically controls FHB otherwise not affected by yeast abundance.

**Figure 4:**
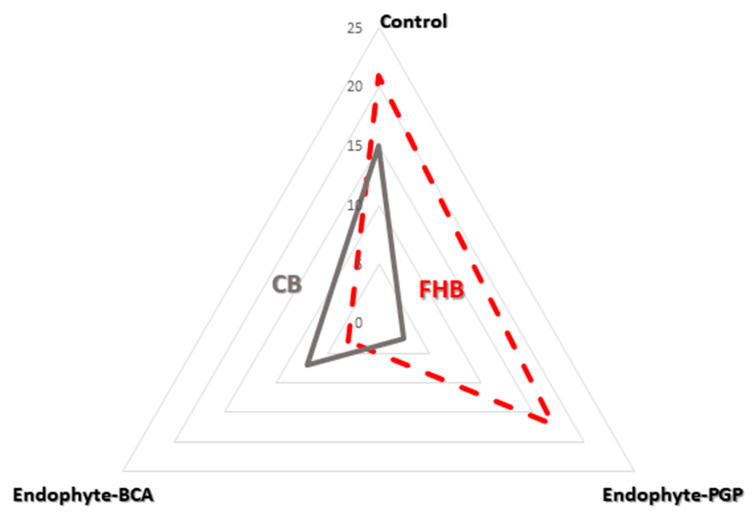
Radar chart displays *Fusarium* (Fusarium head blight) incidence and *Tilletia* (Common bunt/smut) incidence levels (%) in *Triticum* heads. These contain natural *Vishniacozyma* yeasts, which respond to *Penicillium*-endophyte (PGP) and *Sphaerodes*-mycoparasite (BCA) bioinoculations; these represent two ecofriendly ways to protect wheat plants against phytopathogens. The experiment was conducted in five (three plants per pot) replicates under CONVIRON ^®^ PGR15 growth chamber conditions.

Fungal coexistence, rather than antagonism, which many *Vishniacozyma* yeast adopt in relation to some *Rhodosporidiobolus* yeasts, was observed in the studied kernels (Table 1). However, the antagonistic effects of these yeasts resulted in a reduction in the incidence of *Tilletia* and in the CB index (Figure 4). 

## 4. Discussion

There is mounting evidence that fungal endophytes play a vital role in the adaptation of plants to abiotic and biotic stresses. Recently, Vujanovic et al. [2] revealed the importance of transgenerational endophytic fungal diversity in wheat kernels. In that study, Tremellales yeasts were transmitted with Dothideales and Sordariales, as well as the family Ustilentylomataceae, across generations. In addition, the high relative abundance of Tremellales yeasts was consistent with low levels of *Tilletia* (Tilletiales, Exobasidiomycetes) in wheat kernels, which justified further interrelationship studies. 

In this study, we find that wheat kernel harbors several *Vishniacozyma* and *Rhodosporidiobolus* species/genotypes. Interestingly, the presence of multiple yeast taxa in a kernel sample might create a particular microhabitat conductive to hybridization and the eventual emergence of a unique *Vishniacozyma* MT452521 taxon as it is a specific “seminis” to wheat kernel. Eberlain et al. [42] reported that hybridization is a recurrent evolutionary stimulus in wild yeast speciation that adapt to particular environments. The effects of the *Penicillium*-endophyte (PGP) and *Sphaerodes*-mycoparasite (BCA) inoculation on natural Tremellales yeast populations in the kernel were examined in the context of phytoprotection, or improved resistance of wheat to *Tilletia*- and *Fusarium*-caused diseases. Based on both the diversity and changes in relative abundances among members of the yeast consortia in these treatment groups, better prediction and potentially early prevention of biological contamination of wheat kernels appear possible. Indeed, an increased application of both *Penicilllium* and *Sphaerodes* inoculants in the wheat production system could prove beneficial in both enhancing yield and improving kernel health by lowering FHB and CB incidences in treated grain, compared with untreated. This possibility coincides with our previous results, which indicate that a PGP inoculant shifts the microbial composition structure in wheat kernels by increasing Trichocomaceae (e.g., *Penicillium* spp.) and tremelloid-yeast populations [2], which is to the detriment of Exobasidiomycetes (e.g., *Tilletia*) populations. In a synchrotron study conducted by Vujanovic et al. [5], Fourier transform infrared (FTIR) spectroscopy revealed the existence of *Penicillium* endosymbiont-dependent shifts in wheat water economy and nutrient components. Together with the PGP-improved plant biomass, all of these symbiont-related plant traits apparently create an environment in wheat kernel that is conducive to *Vishniacozyma* spp., contributing enhanced plant resilience to *Tilletia*.

Previous Tremellales studies have reported that the protective functions of some yeasts are based on the biosynthesis of defensive metabolites, including extracellular glycolipids and mycocins [38,43,44]. Interestingly, Prior et al. [45] reported that yeasts possess an extensive antagonistic range of defenses against different fungi. According to Ogaki et al. [13], *Vishniacozyma victoriae* was frequently obtained from lichens, mosses, and vascular plants in the arctic environment; however, no antagonism has been detected between *V. victoriae* with other yeasts, and plant pathogens including *F. graminearum.* This phenomenon, which was also observed in the current study, may be partly explained by the commensalism of the yeasts studied. 

The *Sphaerodes*–BCA inoculant has previously shown the ability to promote kernel germination, while providing specific biocontrol against FHB pathogens [31]. In this study, the BCA-specific mycoparasite seemed to compete with yeast mycoparasites by slightly diminishing the efficacy of yeast in suppressing *Tilletia* in wheat kernels (Figure 4; Table 1). Based on these experimental results, we suggest that Tremellomycetes are possible indicators of an altered, competition-driven dynamic among fungi. It also implies a correlation to changes in plant health status induced by external application of endophytic and mycoparasitic inoculants against pathobiota within the kernel ecological niche. This could be partly explained by the niche specialization hypothesis. Indeed, an important level of specificity to the plant host is encrypted in the genome of Tremellales [14], possibly as a result of fungus–host coevolution. Interestingly, the origin of the resistance to *Tilletia* diseases among Canadian wheats, including *T. durum* AC Avonlea, is unknown because there has not been any prior selection for resistance [46]. Considering that tremellomycetous mycoparasites may have coevolved together with their fungal hosts [22], this study emphasizes the possibility that the Tremellomycetous–mycobiome relationship within a wheat host could be best explained by the hologenome theory, which understands the host organism and its microbial symbionts to be a single “holobiont” [47]. In fact, bacterial–fungal associations and communications can form a range of functional interactions within plants, influencing changes in fungal pathogenicity and/or making nutritional influences on the partners and the host fitness [48,49]. In addition, the coexistence and endophytic biocontrol efficacy of microbial consortia are also influenced by signaling pathways between different biocontrol strains [50]. Taking into consideration the holobiont with its hologenome as an independent level of selection, this scientific strategy could possibly make yeasts attractive targets for future studies that focus on their antagonistic relationships toward key pathobiota, which can be validated for practical application by evaluating elite germplasm and mapping microbiome populations. Indeed, emerging expectations are associated with the new biocontrol approaches of using beneficial plant–microbe interactions to enable breeding of microbe-optimized crops [51]. Future integrative research, based on ecosystem ecology of the endophyte-driven wheat genomic and phenotypic treats, could generate new insights. In this context, particular attention can be paid to *Vishniacozyma* spp. yeasts as naturally occurring endophytes, protective mycoparasites, and early bioindicators of the wheat genotype’s resistance to pathobiota.

## 5. Conclusions

This study reports for the first time on the endophytic association of the *Vischniacozymae* species complex and its multitrophic interaction, which involves plant growth-promoting, mycoparasitic, and pathogenic fungi in seed/kernel of a cereal host (*Triticale*). This fungal genus, discovered inside wheat kernels, contains two previously described species (*V.* *victoriae* and *V. tephrensis*), and an undescribed *Vishniacozyma* taxon, as a distinct rDNA variant from the sister clade of *V. carnescens* taxon. The evolutionary rDNA phylogeny indicates its likely emergence through hybridization between diverged wild yeast species/genotypes [52]. This constatation leads to questioning on how an ecological niche affects the coexistence of different yeast genotypes and their functions in plant hosts across environments. These Tremellales yeasts consist predominantly of mycoparasitic or fungicolous taxa. An increased abundance of *Vishniacozyma* yeasts was measured inside kernels of the symbiotic plant host after it acquired an external *Penicillium*–PGP endophyte [5], which also coincided with a reduced CB index. Plant acquisition of the external *Sphaerodes* mycoparasite–BCA inoculant [53] resulted in a reduced FHB index, including *Vishniacozyma* mycoparasitic yeasts. The latter might be interpreted according to the guild hypothesis, which would entail close consideration of the competition between the two mycoparasites within kernel environmental niche. Variations in relative species abundances correlated directly with kernel acquisition of PGP, and indirectly with that of BCA. Both endophytic and biotrophic mycoparasites showed an ability to improve harvested kernel weight/yield. As a reproductive unit of the plant and source of vertical fungal transmission [2], the kernel might be responsible for transgenerational transfer of a structured fungal microbiome that imparts improved plant stress resistance. Since our current understanding is based on the application of individual inoculants, as well as on research under controlled plant growth conditions, further experiments using endophyte–yeast–mycoparasite consortia as kernel coatings are warranted. *Vischniacozyma* spp. merit inclusion as indicators of the microbiome–plant interaction in studies that examine CB and FHB under field conditions, given that YE_index_ values ranged from 0.10 to 0.20 in treated kernel samples. Such future studies can provide deeper insights into practical aspects of yeast application for the purpose of increasing crop production, disease resistance, and improved grain quality. New breeding programs might also profit from host–microbiome compatibility testing that includes promising fungal symbionts, including seed-born *Vischniacozyma* inoculants, and elite wheat germplasm lines under field settings. It appears reasonable to integrate analyses of the genomes of symbionts along with that of the plant host, especially in the context of a “hologenome”, also integrated into a green strategy for next-generation healthy crops.

## Figures and Tables

**Table 1:** Mean values of FHB/CB incidence (DI), disease severity (DS), and FHB and CB indexes for different wheat treatments (Ctr, Endo-PGD, Endo-BCA).

Treatment *	FHB			CB		
	DI (%)	DS(%)	FHB Index	DI (%)	DS (%)	CB Index
Control	21 ^b^	13.8 ^b^	2.9 ^b^	15 ^b^	20.6 ^b^	3.1
Endo-PGP	17 ^ab^	7.1 ^ab^	1.2 ^ab^	2.5 ^a^	0.6 ^a^	0.4 ^a^
Endo-BCA	3.0 ^ab^	3.3 ^ab^	0.1 ^a^	7.0 ^ab^	10 ^ab^	0.7 ^ab^

* within columns, means followed by different letters differ significantly (Turkey’s test, *p* < 0.05).

## Data Availability

All data are presented in this article, while rDNA ITS sequences are submitted, GenBank. accession numbers, in NCBI database. https://www.ncbi.nlm.nih.gov/nucleotide/ (accessed on 30 April 2021).
